# Changes in the Intestinal Microbiome during a Multispecies Probiotic Intervention in Compensated Cirrhosis

**DOI:** 10.3390/nu12061874

**Published:** 2020-06-23

**Authors:** Angela Horvath, Marija Durdevic, Bettina Leber, Katharina di Vora, Florian Rainer, Elisabeth Krones, Philipp Douschan, Walter Spindelboeck, Franziska Durchschein, Gernot Zollner, Rudolf E. Stauber, Peter Fickert, Philipp Stiegler, Vanessa Stadlbauer

**Affiliations:** 1 Department of Gastroenterology and Hepatology, Medical University of Graz, 8036 Graz, Austria; katharina.di-vora@edu.uni-graz.at (K.d.V.); florian.rainer@medunigraz.at (F.R.); elisabeth.krones@medunigraz.at (E.K.); philipp.douschan@medunigraz.at (P.D.); walter.spindelboeck@medunigraz.at (W.S.); franziska.durchschein@medunigraz.at (F.D.); gernot.zollner@medunigraz.at (G.Z.); rudolf.stauber@medunigraz.at (R.E.S.); peter.fickert@medunigraz.at (P.F.); vanessa.stadlbauer@medunigraz.at (V.S.); 2 Center for Biomarker Research in Medicine (CBmed), 8010 Graz, Austria; 3 Center for Medical Research, Medical University of Graz, 8036 Graz, Austria; marija.durdevic@medunigraz.at; 4 Institute of Pathology, Medical University of Graz, 8036 Graz, Austria; 5 Division of Transplantation Surgery, Medical University of Graz, 8036 Graz, Austria; bettina.leber@medunigraz.at (B.L.); philipp.stiegler@medunigraz.at (P.S.)

**Keywords:** gastrointestinal microbiome, RNA, ribosomal, 16S, cirrhosis, probiotics

## Abstract

Probiotics have been used in trials to therapeutically modulate the gut microbiome and have shown beneficial effects in cirrhosis. However, their effect on the microbiome of cirrhosis patients is not fully understood yet. Here, we tested the effects of a multispecies probiotic on microbiome composition in compensated cirrhosis. The gut microbiome composition of 58 patients with compensated cirrhosis from a randomized controlled trial who received a daily dose of multispecies probiotics or placebo for six months was analysed by 16S rRNA gene sequencing. Microbiome composition of patients who received probiotics was enriched with probiotic strains and the abundance of *Faecalibacterium prausnitzii*, *Syntrophococcus sucromutans*, *Bacteroides vulgatus*, *Alistipes shahii* and a *Prevotella* species was increased in the probiotic group compared to the placebo group. Patients who had microbiome changes in response to probiotic treatment also showed a significant increase in neopterin and a significant decrease in faecal zonulin levels after intervention, which was not observed in placebo-treated patients or patients with unchanged microbiome compositions. In conclusion, multispecies probiotics may enrich the microbiome of compensated cirrhotic patients with probiotic bacteria during a six-month intervention and beneficially change the residential microbiome and gut barrier function.

## 1. Introduction

Probiotics is a collective term used for live organisms (usually bacteria or fungi) that can exert health benefits on their host if consumed in sufficient amount [1]. Probiotic bacteria in fermented foods have long been a part of human nutrition but have been neglected in Western style diets [2]. At the same time, the global probiotic market has been booming, as consumers supplement their daily nutrition with products infused with probiotic microorganisms in an effort to nurture their gut microbiota [3]. Medical research has also adopted the idea of using probiotics to modulate the intestinal microbiome of patients in order to achieve a better prognosis [4]. Probiotics are used to prevent antibiotic associated diarrhoea, to treat gastrointestinal diseases, and to reduce the risk for allergies [5,6,7]. In recent reports, however, the use of probiotics came under scrutiny after a controversial study of experimental antibiotic-induced dysbiosis showed a delayed recovery of the autologous microbiome in probiotic-treated test groups. The authors showed that a multispecies probiotic might hinder the re-growth of microbiota by expression of antimicrobial peptides, such as regenerating islet-derived protein 3 gamma (REG3G), and therefore reduce the bacterial load in the intestine [8]. These characteristics were portrayed as the negative end of a trade-off to avoid antibiotic associated diarrhoea. However, in liver cirrhosis, where the microbiome is dominated by the loss of beneficial bacteria and an overgrowth of potential pathogens, which are linked to gut permeability and endotoxemia, a reduction of bacterial load and increase of antimicrobial strategies might be immensely valuable [9,10]. Both the overexpression of Reg3G and the reduction of the intestinal bacterial load showed hepatoprotective characteristics in models of alcoholic liver disease [11,12]. In cirrhosis, the reduction of the intestinal bacterial load is incorporated into clinical practice to treat hepatic encephalopathy and reduce the risk for spontaneous bacterial peritonitis [13]. Aside from the established antibiotic and prebiotic treatments, probiotics have also shown merit in the reduction of hepatic encephalopathy and infections in cirrhosis [14,15,16]. In addition, probiotics can hamper pathogen growth, decrease gut permeability and improve immune responses, which might also be beneficial for patients with cirrhosis [14,17,18,19,20,21,22,23,24,25]. In fact, we have previously shown the beneficial effects of a multispecies probiotic on innate immune reactions and liver function [26]. Although probiotics have been extensively trialled for their effects on clinical outcome parameters, their impact on the composition of the intestinal microbiome is still mostly unexamined. One trial in cirrhotic patients with minimal hepatic encephalopathy showed a reduction in *Enterobacteriaceae* and increase in *Clostridiales Incertae Sedis XIV* and *Lachnospiraceae* abundance [27]. Another trial in decompensated patients with hepatitis B induced cirrhosis showed an increase in *Clostridium I* and *Bifodobacteria*, and a reduction in *Enterobacteriaceae* and *Enterococcus* [28]. However, no such studies have been done in compensated cirrhosis. Therefore, in this study we examined compensated cirrhotic patients before and after a six-month intervention with a multispecies probiotic to test its effect on the composition of the intestinal microbiome.

## 2. Materials and Methods

Stool samples from cirrhosis patients from a previously published randomized, double-blind, placebo controlled study were analysed [26]. Originally, the study was conducted to test the effects of this multispecies probiotic on innate immune function with a distinct focus on neutrophil phagocytosis and oxidative burst, as well as gut permeability and bacterial translocation. There, 92 patients were randomized and 80 patients were analysed (44 in the probiotic group and 36 in the placebo group). This sample size was calculated based on an expected improvement in neutrophil phagocytosis of 25 percentage points and 20% dropout rate with an α of 0.05 and a β of 0.2. During the course of the study, a temporary increase in neopterin and neutrophil resting burst was observed, patients with decompensated cirrhosis showed an improvement in liver function. There were no significant changes in gut permeability or bacterial translocation. The study was conducted between July 2012 and September 2014 at the University Hospital Graz. Stool samples from this study (*n* = 58) were re-evaluated to analyse the effects of the probiotic on microbiome composition. Patients with any type of cirrhosis and willing to give informed consent were included in the present analysis. Patients with a Child-Pugh score of 7 or higher, alcohol abstinence for less than two weeks, clinical evidence for active infection, antibiotic treatment within seven days of enrolment (except for prophylactic treatment of spontaneous bacterial peritonitis), gastrointestinal bleeding within two weeks of enrolment, use of immunomodulating agents, such as steroids within one month of enrolment, concomitant use of pre-, pro- or synbiotics, renal failure with creatinine above 1.7 mg/dL, hepatic encephalopathy stage II or III, pancreatitis, other organ failure, hepatic or extrahepatic malignancies or pregnancy were excluded. As liver function was not well balanced in the original trial and microbiome composition can vary considerably with the severity of liver disease, only patients with Child-Pugh stage A were selected for analysis in the present study. The study was approved by the institutional ethics committee of the Medical University of Graz (23-096 ex 10/11), registered at clinicaltrials.gov (NCT01607528) and performed according to the Declaration of Helsinki. All coauthors had access to the study data and had reviewed and approved the manuscript.Patients received either a six-month intervention with a daily dose of a multispecies preparation containing 1.5 × 0^10^ colony forming units in 6 g of powder or a similarly tasting placebo, which consisted of the matrix without bacteria. The product consists of *Bifidobacterium bifidum* W23, *Bifidobacterium lactis* W51, *Bifidobacterium lactis* W52, *Lactobacillus acidophilus* W37, *Lactobacillus brevis* W63, *Lactobacillus casei* W56, *Lactobacillus salivarius* W24, *Lactococcus lactis* W19 and *Lactococcus lactis* W58 in a matrix of maize starch, maltodextrins, vegetable protein, potassium chloride, magnesium sulphate, enzymes (amylases) and manganese sulphate. These strains were selected because they showed beneficial effects to varying degrees on strengthening epithelial monolayers after an infectious as well as an inflammatory stressor, inhibiting mast cell activation, stimulating IL10 expression and decreasing endotoxin load in vitro [21]. This product is marketed as Ecologic^®^ Barrier in The Netherlands (Winclove, Amsterdam, The Netherlands) and as Omni-Biotic Hetox in Austria, Germany and Switzerland (Institut Allergosan, Graz, Austria). The study product was kept in consecutively numbered but otherwise blank sachets, so that patients, caregivers, investigators and outcome assessors were blinded to their content. Randomization was done by permutated blocks in a 1:1 ratio using Randomizer^®^ software Version 1.8.2/1.8.3 (Institute of Medical Informatics, Medical University of Graz, Graz, Austria).

Participants were asked to complete a self-administered food frequency questionnaire to document possible changes in dietary habits. All clinical parameters were taken from a previously published study and methodology is described there [26]. Physical activity was not controlled during the trial.

Before the intervention (Baseline), immediately after the end of intervention (Intervention/Placebo) and after a six-month follow up period (Observation), stool samples were collected and stored in sterile collection tubes at −80 °C. After all patients finished the study, DNA was isolated from the stool samples with the MagNA Pure LC DNA Isolation Kit III (Bacteria, Fungi) (Roche, Mannheim, Germany) according to manufacturer’s instructions. Hypervariable region V1-V2 was amplified (primers: 27F-AGAGTTTGATCCTGGCTCAG; R357-CTGCTGCCTYCCGTA) and sequenced using Illumina Miseq technology (Illumina, Eindhoven, The Netherlands), as published before [29]. These sequence data have been submitted to NCBI Sequence Read Archive databases under accession number SRP132827.

Data from 58 patients at the three defined time points was analysed using QIIME 2 tools on a local Galaxy instance (https://galaxy.medunigraz.at/) [30]. Denoising (primers removing, quality filtering, correcting errors in marginal sequences, removing chimeric sequences, removing singletons, joining paired-end reads, and dereplication) was done with DADA2 [31]. Taxonomy was assigned based on Silva 132 database release at 99% operational taxonomic unit level with a Naïve Bayes classifier.

Microbiome analysis was performed on a non-summarized operational taxonomic unit (OTU) level. Chao1 index was used to quantify alpha diversity. A Bray Curtis dissimilarity matrix was the basis for redundancy analysis (RDA), non-metric multidimensional scaling (NMDS) and analysis of similarity (ANOSIM), all done in Calypso 7.14 (http://cgenome.net/calypso/). Gneiss differential abundance analysis and analysis of composition of microbiomes (ANCOM) statistics from the q2-composition plug-in implemented in QIIME2 were used to compare OTU abundances. Differential abundance analysis was based on correlation clustering (gneiss correlation-clustering) where Ward hierarchical clustering was used to cluster groups of OTUs together that were correlated with each other. Isometric log ratios were calculated and compared for subsets of taxa in order to obtain balances. At the end, the most statistically significant balances were examined to define what taxa constitute those partitions. Associations between the microbiome and clinical parameters were evaluated with Spearman-correlations and between-group differences were assessed with Kruskal-Wallis test and Bonferroni multiplicity correction, performed in SPSS for Windows Version 23 (SPSS Inc., Chicago, IL, USA). *p*-value < 0.05 was considered to be statistically significant. Visualization was done in GraphPad Prism 6 (GraphPad Software, San Diego, CA, USA).

## 3. Results

**Figure 1:**
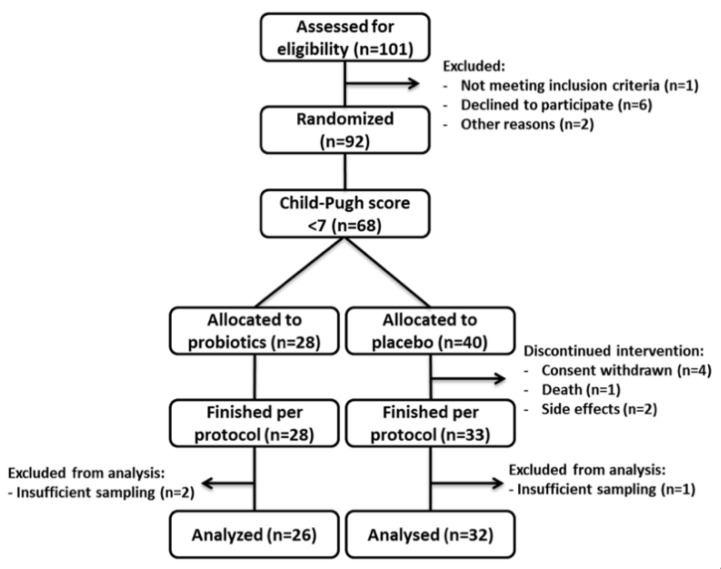
Enrolment scheme.

For this study, patients with compensated cirrhosis were selected from a well characterized cohort that underwent a six-month probiotic intervention and were then followed up for another six months without intervention [26]. In total, 58 patients were analysed, 26 were allocated to the probiotic group and 32 to the placebo group. Patients’ characteristics are given in Table 1, enrolment is summarized in Figure 1.

In total 10.998.425 and 14.107.620 sequencing reads were analysed in the probiotic and placebo group, respectively. After quality checking and denoising, the number of sequences was reduced to 6.967.396 in probiotic and 8.947.492 in placebo group. Around 17% of the reads in each group were not merged during the merging paired-end reads step. Approximately 20% of sequences in each group were chimera and therefore removed. Ultimately, 4.616.745 and 5.834.621 sequences per group entered the taxonomic assignment step, and an average of 60.746 ± 15.792 and 61.417 ± 30.305 sequences per sample were analysed in the probiotic and placebo group, respectively. Since diet is a potential confounder in the analysis of microbiome composition, patients’ nutrition was monitored with a 36-item food frequency questionnaire; no changes in nutrition were observed.

### 3.1. Alpha and Beta Diversity

**Figure 2:**
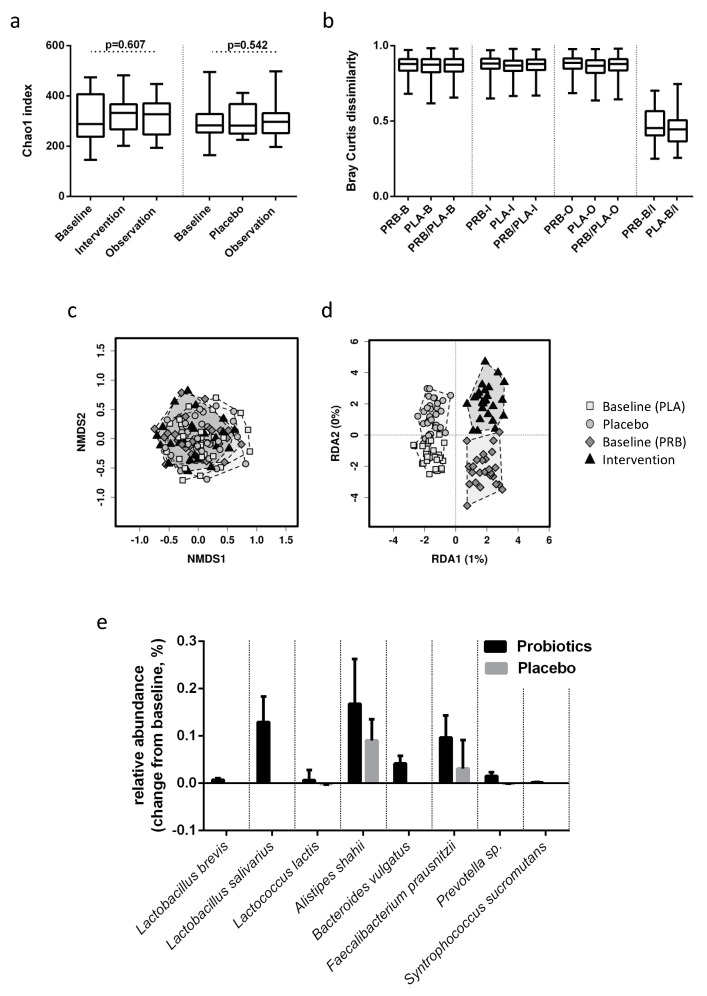
Probiotic modulation of the microbiome in compensated cirrhosis. (**a**) Chao1 index for probiotic- and placebo-treated patients before and after intervention and after follow up. (**b**) Bray Curtis dissimilarities within and between probiotic-treated (PRB) and placebo-treated (PLA) patients at baseline (B), after intervention (I) and at follow up (O). (**c**) Non-metric multidimensional scaling plot of probiotic (PRB)- and placebo (PLA)- treated patients before (Baseline) and after intervention (Intervention/Placebo). (**d**) Redundancy analysis for intervention and time point. (**e**) Differentially abundant OTUs at the end of intervention presented as changes from baseline in relative abundance for probiotic and placebo-treated patients. Bars represent mean and standard error.

Alpha diversity did not differ significantly between probiotic and placebo treated patients before (Chao1: 317 ± 96 vs. 287 ± 70, respectively, *p* = 0.407) or after the intervention (326 ± 72 vs. 306 ± 59, respectively, *p* = 0.521). There were no significant changes over time within the groups (Figure 2a).

Beta diversity remained stable throughout the intervention in both subject groups. Bray Curtis dissimilarity showed that microbiome compositions were comparable in both groups before and after intervention. The intra-individual distance between samples before and after intervention was similar in both groups, and significantly lower than the distance to other individuals (Figure 2b). Accordingly, NMDS based on Bray Curtis dissimilarity and consecutive ANOSIM revealed no specific clustering of groups or time points (*p* = 0.516), as shown in Figure 2c. RDA showed no significant correlation between the overall microbiome composition and probiotic intervention *p* > 0.999 (Figure 2d).

### 3.2. Taxon Comparison

Changes in unique OTUs were analysed using ANCOM and differential abundance analysis with Gneiss and were deemed of interest if a significant difference was observed between baseline and end of intervention as well as between groups at the end of treatment. While ANCOM did not show any differences between groups or timepoints, 11 out of 16,032 observed OTUs met these criteria when using differential abundance analysis with Gneiss in the probiotic group and none in the placebo group.

Three differentially abundant OTUs were identical with the sequences of the probiotic bacteria *Lactobacillus salivarius* W24, *Lactobacillus brevis* W63 and *Lactococcus lactis* W19/W58, which were ingested with the study product. Those OTUs were more abundant after the intervention in the probiotic group, while patients in the placebo group showed little to no abundance. In the probiotic group, increased levels were detected only immediately after the intervention, not at baseline or six months after the intervention had ended (Figure 2e). The remaining probiotic strains ingested with the study product were not indicated in the a priori analysis. A separate search for these sequences showed that *Bifidobacterium bifidum*, *Lactobacillus acidophilus* and *Lactobacillus casei* remained on their endogenous levels, *Bifidobacterium lactis* could not be found.

Furthermore, an OTU identified as *Faecalibacterium prausnitzii* was significantly increased by the probiotic intervention, resulting in a significantly higher abundance after six months compared to the placebo group. It almost doubled in abundance and remained elevated after the intervention had ended. OTUs identified as *Syntrophococcus sucromutans*, *Bacteroidetes vulgatus* and *Prevotella* sp. showed steep increases during intervention and retreated after the intervention had ended. *Alistipes shahii* increased in abundance during intervention and further increased after intervention had ended (Figure 2e).

*Ruminococcaceae UCG-014* decreased after the intervention and regained baseline levels six months after the intervention had ended. However, a drop in abundance of this taxon was also observed in the placebo group. A slight decrease in abundance of *Anaerostipes hadrus* was observed in the probiotic group, but this OTU was absent in the placebo group before and after intervention. *Dorea longicatena* was also identified as significantly altered; however, it was only present in four samples (two samples in the probiotic group before intervention and two samples in the placebo group after intervention). Intervention-independent longitudinal changes were observed that mainly included the increase or decrease of various sequences identified as *Bacteroides vulgatus*, a starch-utilizing bacterium.

### 3.3. Associations with Clinical Findings

**Figure 3:**
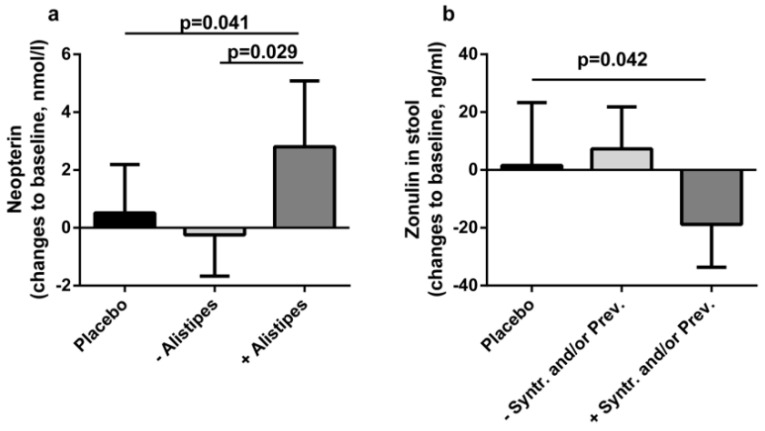
Associations with clinical outcome. (**a**) Changes in serum neopterin levels from baseline to the end of intervention of patients with increased *Alistipes* abundance (+), decreased or unchanged abundance (−), or placebo-treated patients. (**b**) Changes in faecal zonulin levels of patients with increased *Syntrophococcus* and or *Prevotella* abundance (+), decreased or unchanged abundance (−), or placebo-treated patients.

To assess clinical significance of the observed changes in the microbiome at the end of intervention, their associations with biomarkers of gut permeability (faecal zonulin, lactulose-mannitol ratio, and diamine oxidase), bacterial translocation (lipopolysaccharide, sCD14, and lipopolysaccharide binding protein) and innate immune response (neopterin, and resting burst by neutrophils) were examined. Correlation analysis associated changes in neopterin to changes in *Alistipes shahii* abundance (r_s_ = 0.354; *p* = 0.006), and changes in zonulin to changes in *Syntrophococcus sucromutans* and *Prevotella* sp. (r_s_ = −0.311; *p* = 0.018 and r_s_ = −0.285; *p* = 0.030, respectively). Accordingly, patients with an increase in *Alistipes shahii* after probiotic intervention show a significant increase in neopterin compared to placebo-treated patients and patients without that bacterial increase. Moreover, patients with an increase in *Syntrophococcus sucromutans* and/or *Prevotella* sp. showed a significant decrease in zonulin compared to placebo-treated patients (Figure 3). Other biomarkers showed no significant correlation to the identified OTUs.

## 4. Discussion

Our data show that after six months of probiotic intervention the probiotic strains *Lactobacillus brevis*, *Lactobacillus salivarius* and *Lactococcus lactis* were enriched in the stool of cirrhotic patients and the abundance of *Faecalibacterium prausnitzii*, *Syntrophococcus sucromutans* and *Alistipes shahii* (all short-chain acid producers), as well as *Bacteroides vulgatus*, and a *Prevotella* species were increased in the probiotic group at the end of the intervention. Changes in the microbiome correlated with changes in serum neopterin levels and gut permeability.

When efficacy of probiotic therapies is discussed, it is necessary to know whether the probiotic preparation in question can reach the intended target in the intestine. Probiotic colonization shows a very high inter-individual variability and the resident microbiome can oppose the introduction of exogenous bacteria [32]. Cirrhotic patients show a significantly reduced microbial richness and therefore a reduced colonization resistance [10,33,34]. This could lead to a more efficient integration of probiotic bacteria into the resident microbiome [8]. In liver cirrhosis, an increase in the abundance of probiotic bacteria after ingestion has so far only been shown in patients with decompensated disease, where probiotic bacteria may find more favourable conditions for colonization due to severe dysbiosis [27,28]. In the present study, we demonstrated the transient enrichment of probiotic bacteria in compensated cirrhosis. Previous reports have shown that the enrichment of the microbiome with probiotic strains is dependent on ongoing administration [35,36,37] and our data confirm this, since we show that the abundance of probiotic bacteria regresses after probiotic therapy has been completed.

Probiotic intervention in our study was not associated with monumental changes in the composition of the resident microbiome whereas in trials with decompensated patients, changes in major taxa of the human microbiome, such as an increase in *Clostridiales* or a reduction in *Enterobacteriaceae* with probiotic therapy were observed [27,28]. Changes observed in the present study were only found on OTU level and no major taxon significantly shifted in abundance. A possible reason for the different results of these studies might be found in the underlying health condition of the study cohorts. Compared to compensated cirrhosis, the microbiome of patients with decompensated cirrhosis is less stable due to various factors, such as medical treatment and hospitalization [9]. Therefore, probiotics might be able to induce larger changes in decompensated cirrhosis; however, in relatively stable conditions, big shifts in composition might not be achieved as easily.

Whether small changes in the microbiome are sufficient to induce clinical benefits remains unclear. We found correlations between the modulation of *Alistipes shahii* and the increase in neopterin, a macrophage-derived antimicrobial molecule that can induce reactive oxygen species production by neutrophils [38,39]. This increase in antimicrobial strategies is consistent with previous reports that attest antimicrobial properties to probiotic strains or a boost of host derived immune responses to their ingestion [8,25,40,41,42,43,44]. Moreover, *Alistipes* species were found to be depleted in patients with liver cirrhosis and non-alcoholic fatty liver disease [10,45]. An increase of *Alistipes* might signify an opposition to liver disease specific dysbiosis.

The increase of OTUs attributed to *Synthrophococcus* and *Prevotella* were correlated to a decrease in zonulin, an endogenous tight-junction regulating protein and is used as a biomarker for gut permeability, where high levels in stool or serum are indicative of a barrier dysfunction [46,47]. Decrease in zonulin levels during probiotic interventions has been shown before in obese patients with type 2 diabetes with the same product as used in the present study, and in healthy trained men with a comparable multispecies product [23,48]. Furthermore, the same product as in the presented trial showed a reduction of bacterial translocation in type 2 diabetic patients after a six month intervention and in postmenopausal women after 12 weeks [49,50]. Our results suggest that the improvement of the gut barrier is dependent on intervention-related modulation of the microbiome. Further, improvements in clinical outcomes in other diseases might be linked to successful modulation of the microbiome [32,51].

Besides the intervention-specific changes in the microbiome, OTUs that were identified as *Bacteroides vulgatus* showed fluctuations throughout the study in both groups. *Bacteroides vulgatus* utilizes starch as a primary substrate [52], and since these changes in abundance are prevalent in both groups, it is possible that this reflects a reaction of the microbiome to the product matrix, which consisted primarily of maize starch and was the sole ingredient in the placebo. Reactions to a product matrix should be a focus in future studies and considered in the product development.

Limitations: Although the analysed samples were from a randomized, double blind, placebo-controlled study, the inclusion in this subgroup analysis was based on liver disease severity. Accounting for the significant differences in the probiotic modulation of the microbiome between compensated and decompensated cirrhosis and the instability of the microbiome during decompensation at the same time would require a robust control group. Due to uneven dropout rates, such a control group was not available and patients with decompensated cirrhosis could not be selected for this analysis.

Furthermore, it should be noted that the differences in microbiome composition obtained by one method could not be reproduced by other statistical tools. Although the methods are not identical, they were both optimized for the identification of differentially abundant OTUs in compositional data. The controversial nature of their results might simply indicate that they are based on different algorithms and weigh specific characteristics of the data set differently. However, they might also indicate that the results are not robust enough to be identified by more than one method and need to be reproduced in future studies. Therefore, we encourage the careful deliberation of the results before interpretation.

## 5. Conclusions

After a six-month intervention with probiotics, an increase in probiotic bacteria and other beneficial taxa in stool of compensated cirrhotic patients was evident. Most of these changes were not permanent, suggesting that ongoing administration might be necessary to achieve long-term effects in this patient cohort. Further studies are necessary to elucidate the clinical relevance of microbiome changes.

## Figures and Tables

**Table 1:** Baseline characteristics of patients according to allocation; data is presented as mean (±standard deviation) or count.

Characteristic	Probiotics (*n* = 26)	Placebo (*n* = 32)	*p*-Value
Age	60 (±7)	55 (±10)	0.08
Gender (m/f)	17/9	23/9	0.78
Child-Pugh score (5/6)	20/6	24/8	>0.99
MELD ^1^ score	10 (±3)	10 (±3)	0.88
PPI ^2^ use (yes/no)	15/11	15/17	0.44
Beta blocker (yes/no)	14/12	16/16	0.77
Antibiotic use (yes/no)	0/26	0/32	-

^1^ Model of End-stage Liver Disease; ^2^ Proton pump inhibitor.

## References

[1] Sanders M.E. (2008). Probiotics: Definition, sources, selection, and uses. Clin. Infect. Dis..

[2] Chilton S.N., Burton J.P., Reid G. (2015). Inclusion of fermented foods in food guides around the world. Nutrients.

[3] Sanders M.E. (2015). Probiotics in 2015: Their Scope and Use. J. Clin. Gastroenterol..

[4] Islam S.U. (2016). Clinical Uses of Probiotics. Medicine.

[5] Floch M.H., Walker W.A., Madsen K., Sanders M.E., Macfarlane G.T., Flint H.J., Dieleman L.A., Ringel Y., Guandalini S., Kelly C.P. (2011). Recommendations for probiotic use—2011 update. J. Clin. Gastroenterol..

[6] Dotterud C.K., Storro O., Johnsen R., Oien T. (2010). Probiotics in pregnant women to prevent allergic disease: A randomized, double-blind trial. Br. J. Dermatol..

[7] Wickens K., Black P.N., Stanley T.V., Mitchell E., Fitzharris P., Tannock G.W., Purdie G., Crane J. (2008). A differential effect of 2 probiotics in the prevention of eczema and atopy: A double-blind, randomized, placebo-controlled trial. J. Allergy Clin. Immunol..

[8] Suez J., Zmora N., Zilberman-Schapira G., Mor U., Dori-Bachash M., Bashiardes S., Zur M., Regev-Lehavi D., Ben-Zeev Brik R., Federici S. (2018). Post-Antibiotic Gut Mucosal Microbiome Reconstitution Is Impaired by Probiotics and Improved by Autologous FMT. Cell.

[9] Bajaj J.S., Heuman D.M., Hylemon P.B., Sanyal A.J., White M.B., Monteith P., Noble N.A., Unser A.B., Daita K., Fisher A.R. (2014). The Cirrhosis Dysbiosis Ratio defines Changes in the Gut Microbiome Associated with Cirrhosis and its Complications. J. Hepatol..

[10] Qin N., Yang F., Li A., Prifti E., Chen Y., Shao L., Guo J., Le Chatelier E., Yao J., Wu L. (2014). Alterations of the human gut microbiome in liver cirrhosis. Nature.

[11] Wang L., Fouts D.E., Starkel P., Hartmann P., Chen P., Llorente C., DePew J., Moncera K., Ho S.B., Brenner D.A. (2016). Intestinal REG3 Lectins Protect against Alcoholic Steatohepatitis by Reducing Mucosa-Associated Microbiota and Preventing Bacterial Translocation. Cell Host Microbe.

[12] Adachi Y., Moore L.E., Bradford B.U., Gao W., Thurman R.G. (1995). Antibiotics prevent liver injury in rats following long-term exposure to ethanol. Gastroenterology.

[13] European Association for the Study of the Liver (2018). Clinical Practice Guidelines for the management of patients with decompensated cirrhosis. J. Hepatol..

[14] Liu Q., Duan Z.P., Ha D.K., Bengmark S., Kurtovic J., Riordan S.M. (2004). Synbiotic modulation of gut flora: Effect on minimal hepatic encephalopathy in patients with cirrhosis. Hepatology.

[15] Rayes N., Seehofer D., Theruvath T., Schiller R.A., Langrehr J.M., Jonas S., Bengmark S., Neuhaus P. (2005). Supply of pre- and probiotics reduces bacterial infection rates after liver transplantation—A randomized, double-blind trial. Am. J. Transplant..

[16] Sawas T., Al Halabi S., Hernaez R., Carey W.D., Cho W.K. (2015). Patients Receiving Prebiotics and Probiotics Before Liver Transplantation Develop Fewer Infections Than Controls: A Systematic Review and Meta-Analysis. Clin. Gastroenterol. Hepatol..

[17] Lata J., Novotny I., Pribramska V., Jurankova J., Fric P., Kroupa R., Stiburek O. (2007). The effect of probiotics on gut flora, level of endotoxin and Child-Pugh score in cirrhotic patients: Results of a double-blind randomized study. Eur. J. Gastroenterol. Hepatol..

[18] Kirpich I.A., Solovieva N.V., Leikhter S.N., Shidakova N.A., Lebedeva O.V., Sidorov P.I., Bazhukova T.A., Soloviev A.G., Barve S.S., McClain C.J. (2008). Probiotics restore bowel flora and improve liver enzymes in human alcohol-induced liver injury: A pilot study. Alcohol.

[19] Johansson M.L., Molin G., Jeppsson B., Nobaek S., Ahrne S., Bengmark S. (1993). Administration of different Lactobacillus strains in fermented oatmeal soup: In vivo colonization of human intestinal mucosa and effect on the indigenous flora. Appl. Environ. Microbiol..

[20] Lidbeck A., Gustafsson J.A., Nord C.E. (1987). Impact of *Lactobacillus acidophilus* supplements on the human oropharyngeal and intestinal microflora. Scand. J. Infect. Dis..

[21] Van Hemert S., Ormel G. (2014). Influence of the Multispecies Probiotic Ecologic^®^ BARRIER on Parameters of Intestinal Barrier Function. Food Nutr. Sci..

[22] Chen P., Torralba M., Tan J., Embree M., Zengler K., Starkel P., van Pijkeren J.P., DePew J., Loomba R., Ho S.B. (2015). Supplementation of saturated long-chain fatty acids maintains intestinal eubiosis and reduces ethanol-induced liver injury in mice. Gastroenterology.

[23] Lamprecht M., Bogner S., Schippinger G., Steinbauer K., Fankhauser F., Hallstroem S., Schuetz B., Greilberger J.F. (2012). Probiotic supplementation affects markers of intestinal barrier, oxidation, and inflammation in trained men; a randomized, double-blinded, placebo-controlled trial. J. Int. Soc. Sports Nutr..

[24] Sultana R., McBain A.J., O’Neill C.A. (2013). Strain-dependent augmentation of tight-junction barrier function in human primary epidermal keratinocytes by *Lactobacillus* and *Bifidobacterium* lysates. Appl. Environ. Microbiol..

[25] Stadlbauer V., Mookerjee R.P., Hodges S., Wright G.A., Davies N.A., Jalan R. (2008). Effect of probiotic treatment on deranged neutrophil function and cytokine responses in patients with compensated alcoholic cirrhosis. J. Hepatol..

[26] Horvath A., Leber B., Schmerboeck B., Tawdrous M., Zettel G., Hartl A., Madl T., Stryeck S., Fuchs D., Lemesch S. (2016). Randomised clinical trial: The effects of a multispecies probiotic vs. placebo on innate immune function, bacterial translocation and gut permeability in patients with cirrhosis. Aliment. Pharmacol. Ther..

[27] Bajaj J.S., Heuman D.M., Hylemon P.B., Sanyal A.J., Puri P., Sterling R.K., Luketic V., Stravitz R.T., Siddiqui M.S., Fuchs M. (2014). Randomised clinical trial: *Lactobacillus* GG modulates gut microbiome, metabolome and endotoxemia in patients with cirrhosis. Aliment. Pharmacol. Ther..

[28] Xia X., Chen J., Xia J., Wang B., Liu H., Yang L., Wang Y., Ling Z. (2018). Role of probiotics in the treatment of minimal hepatic encephalopathy in patients with HBV-induced liver cirrhosis. J. Int. Med. Res..

[29] Stadlbauer V., Horvath A., Ribitsch W., Schmerbock B., Schilcher G., Lemesch S., Stiegler P., Rosenkranz A.R., Fickert P., Leber B. (2017). Structural and functional differences in gut microbiome composition in patients undergoing haemodialysis or peritoneal dialysis. Sci. Rep..

[30] Caporaso J.G., Kuczynski J., Stombaugh J., Bittinger K., Bushman F.D., Costello E.K., Fierer N., Pena A.G., Goodrich J.K., Gordon J.I. (2010). QIIME allows analysis of high-throughput community sequencing data. Nat. Methods.

[31] Callahan B.J., McMurdie P.J., Rosen M.J., Han A.W., Johnson A.J., Holmes S.P. (2016). DADA2: High-resolution sample inference from Illumina amplicon data. Nat. Methods.

[32] Zmora N., Zilberman-Schapira G., Suez J., Mor U., Dori-Bachash M., Bashiardes S., Kotler E., Zur M., Regev-Lehavi D., Brik R.B. (2018). Personalized Gut Mucosal Colonization Resistance to Empiric Probiotics Is Associated with Unique Host and Microbiome Features. Cell.

[33] Case T.J. (1990). Invasion resistance arises in strongly interacting species-rich model competition communities. Proc. Natl. Acad. Sci. USA.

[34] He X., McLean J.S., Guo L., Lux R., Shi W. (2014). The social structure of microbial community involved in colonization resistance. ISME J..

[35] Bouhnik Y., Pochart P., Marteau P., Arlet G., Goderel I., Rambaud J.C. (1992). Fecal recovery in humans of viable *Bifidobacterium* sp. ingested in fermented milk. Gastroenterology.

[36] Kullen M.J., Amann M.M., O’Shaughnessy M.J., O’Sullivan D.J., Busta F.F., Brady L.J. (1997). Differentiation of ingested and endogenous bifidobacteria by DNA fingerprinting demonstrates the survival of an unmodified strain in the gastrointestinal tract of humans. J. Nutr..

[37] Goldin B.R., Gorbach S.L., Saxelin M., Barakat S., Gualtieri L., Salminen S. (1992). Survival of *Lactobacillus* species (strain GG) in human gastrointestinal tract. Dig. Dis. Sci..

[38] Hoffmann G., Wirleitner B., Fuchs D. (2003). Potential role of immune system activation-associated production of neopterin derivatives in humans. Inflamm. Res..

[39] Razumovitch J.A., Fuchs D., Semenkova G.N., Cherenkevich S.N. (2004). Influence of neopterin on generation of reactive species by myeloperoxidase in human neutrophils. Biochim. Biophys. Acta.

[40] Bertuccini L., Russo R., Iosi F., Superti F. (2017). Effects of *Lactobacillus rhamnosus* and *Lactobacillus acidophilus* on bacterial vaginal pathogens. Int. J. Immunopathol. Pharmacol..

[41] Marianelli C., Cifani N., Pasquali P. (2010). Evaluation of antimicrobial activity of probiotic bacteria against *Salmonella enterica* subsp. *enterica* serovar typhimurium 1344 in a common medium under different environmental conditions. Res. Microbiol..

[42] Wan M.L.Y., Forsythe S.J., El-Nezami H. (2019). Probiotics interaction with foodborne pathogens: A potential alternative to antibiotics and future challenges. Crit. Rev. Food Sci. Nutr..

[43] Azad M.A.K., Sarker M., Wan D. (2018). Immunomodulatory Effects of Probiotics on Cytokine Profiles. Hindawi.

[44] Delcenserie V., Martel D., Lamoureux M., Amiot J., Boutin Y., Roy D. (2008). Immunomodulatory effects of probiotics in the intestinal tract. Curr. Issues Mol. Biol..

[45] Jiang W., Wu N., Wang X., Chi Y., Zhang Y., Qiu X., Hu Y., Li J., Liu Y. (2015). Dysbiosis gut microbiota associated with inflammation and impaired mucosal immune function in intestine of humans with non-alcoholic fatty liver disease. Sci. Rep..

[46] Fasano A. (2012). Intestinal permeability and its regulation by zonulin: Diagnostic and therapeutic implications. Clin. Gastroenterol. Hepatol..

[47] Sturgeon C., Lan J., Fasano A. (2017). Zonulin transgenic mice show altered gut permeability and increased morbidity/mortality in the DSS colitis model. Ann. N Y Acad. Sci..

[48] Horvath A., Leber B., Feldbacher N., Tripolt N., Rainer F., Blesl A., Trieb M., Marsche G., Sourij H., Stadlbauer V. (2019). Effects of a multispecies synbiotic on glucose metabolism, lipid marker, gut microbiome composition, gut permeability, and quality of life in diabesity: A randomized, double-blind, placebo-controlled pilot study. Eur. J. Nutr..

[49] Sabico S., Al-Mashharawi A., Al-Daghri N.M., Wani K., Amer O.E., Hussain D.S., Ansari M.G.A., Masoud M.S., Alokail M.S., McTernan P.G. (2018). Effects of a 6-month multi-strain probiotics supplementation in endotoxemic, inflammatory and cardiometabolic status of T2DM patients: A randomized, double-blind, placebo-controlled trial. Clin. Nutr..

[50] Szulinska M., Loniewski I., van Hemert S., Sobieska M. (2018). Dose-Dependent Effects of Multispecies Probiotic Supplementation on the Lipopolysaccharide (LPS) Level and Cardiometabolic Profile in Obese Postmenopausal Women: A 12-Week Randomized Clinical Trial. Nutrients.

[51] Zhao L., Zhang F. (2018). Gut bacteria selectively promoted by dietary fibers alleviate type 2 diabetes. Science.

[52] McCarthy R.E., Pajeau M., Salyers A.A. (1988). Role of starch as a substrate for *Bacteroides vulgatus* growing in the human colon. Appl. Environ. Microbiol..

